# Supplementary material for: Expression, refolding and spectroscopic characterization of fibronectin type III (FnIII)-homology domains derived from human fibronectin leucine rich transmembrane protein (FLRT)-1, -2, and -3
Source: PeerJ. 2017 Jul 7;5:e3550. doi: 10.7717/peerj.3550 (PMC5502089; doi:10.7717/peerj.3550)
Supplement: Supplemental Information 1 [file peerj-05-3550-s001.docx]

**Supplemental Information**

**Expression, refolding and spectroscopic characterization of Fibronectin type III (FnIII)-homology domains derived from human Fibronectin Leucine Rich Transmembrane Protein (FLRT)-1, -2, and -3**

Lila Yang^1§^, Maria Hansen Falkesgaard^2§^, Peter Waaben Thulstrup^1^, Peter Schledermann Walmod^2^, Leila Lo Leggio^1^ and Kim Krighaar Rasmussen^1,2^

^1^ Biological Chemistry, Department of Chemistry, University of Copenhagen, Copenhagen, Denmark

^2^ Laboratory of Neural Plasticity, Department of Neuroscience and Pharmacology, Faculty of Health and Medical Sciences, University of Copenhagen, Copenhagen, Denmark

^§^ These authors have contributed equally

Corresponding author

*Kim Krighaar Rasmussen*^1^

Universitetsparken 5, Copenhagen, Denmark, 2100, Denmark

*KKR@chem.ku.dk*

*Leila Lo Leggio*^1^

Universitetsparken 5, Copenhagen, Denmark, 2100, Denmark

*Leila@chem.ku.dk*

Table S1

|  | M_wobs_ | M_wtheo._ | ∆_mass_ | Fragment |
| --- | --- | --- | --- | --- |
| FLRT1-FnIII | 11122.8 | 11125.560 | 2.759 | **HVKALTADSIRITWKATLASSFRLSWLRLGHSPAVGSITETLVQGDKTEYLLTALEPKSTYIICMVTMETSNAYVADETPVCAKAETAD SYGPTHHHHHH** |
| FLRT1-FnIII | 11254.2 | 11254.639 | 0.438 | **IHVKALTADSIRITWKATLASSFRLSWLRLGHSPAVGSITETLVQGDKTEYLLTALEPKSTYIICMVTMETSNAYVADETPVCAKAETAD SYGPTHHHHHH** |
| FLRT1-FnIII | 11947.6 | 11952.015 | 4.414 | **GDGAKTLAIHVKALTADSIRITWKATLASSFRLSWLRLGHSPAVGSITETLVQGDKTEYLLTALEPKSTYIICMVTMETSNAYVADETPVCAKAETADSYGPTHHHHHH** |
| FLRT1-FnIII | 12077.3 | 12073.72 | 3.58 | **MGDGAKTLAIHVKALTADSIRITWKATLASSFRLSWLRLGHSPAVGSITETLVQGDKTEYLLTALEPKSTYIICMVTMETSNAYVADETPVCAKAETAD SYGPTHHHHHH** |
| FLRT2-FnIII | 11746.3 | 11745.989 | -0.311 | **RIQLSIHFVNDTSIQVSWLSLFTVMAYKLTWVKMGHSLVGGIVQERIVSGEKQHLSLVNLEPRSTYRICLVPLDAFNYRAVEDTICSEATTHASYLHHHHHH** |
| FLRT3-FnIII | 12230.8 | 12234.199 | 3.398 | **GSPSRKTITITVKSVTSDTIHISWKLALPMTALRLSWLKLGHSPAFGSITETIVTGERSEYLVTALEPDSPYKVCMVPMETSNLYLFDETPVCIETETAPLRMHHHHHH** |

Analysis of masses measured with MALDI-TOF. The measured samples obtained from MALDI-TOF, were together with the sequence of protein fed to the FindPept at Expasy.org. FindPept was allowed to freely oxidize methionine without limitations. Underlined M means oxidized methionine.

Table S2

| Structure prediction of FnIIIs | C-score | TM-Score |  |
| --- | --- | --- | --- |
| FLRT1 model 1 | -0.45 | 0.66+-0.13 |  |
| FLRT2 model 1 | -0.13 | 0.70 +- 0.12 |  |
| FLRT3 model 1 | -0.37 | 0.67+- 0.13 |  |

The top models from I-TASSER have been listed, together with validation scores (C-score and TM-score) The C-score is in the range -5,2 and a model with C-score >-1.5 and a with a TM-score > 0.5 usually has a correct fold. TM-score is independent of sequences length, and is a measure for structure similarity with a value in the range 0,1. The models given in the table, are the models presented in Figure 5.

Figure S1

The folding strategy was confirmed from ^1^H-NMR spectrum of FLRT1-FnIII. Chemical shifts arise due to small variation in the local chemical environment surrounding the amino acid residue. An unfolded protein would have similar H-N shift, due to similar environmental surroundings, and the spectra collapse around 8.2 ppm. As seen a broad dispersion of peaks is observed, indicating folded protein. Furthermore, the two peaks W1 and W2 are chemical shift (H-Nε) from the two tryptophan residues in FLRT1-FnIII. This shows that the two tryptophans in FLRT1-FnIII have distinct local environment, and are strong indication that the FnIII domain is folded.

Figure S2


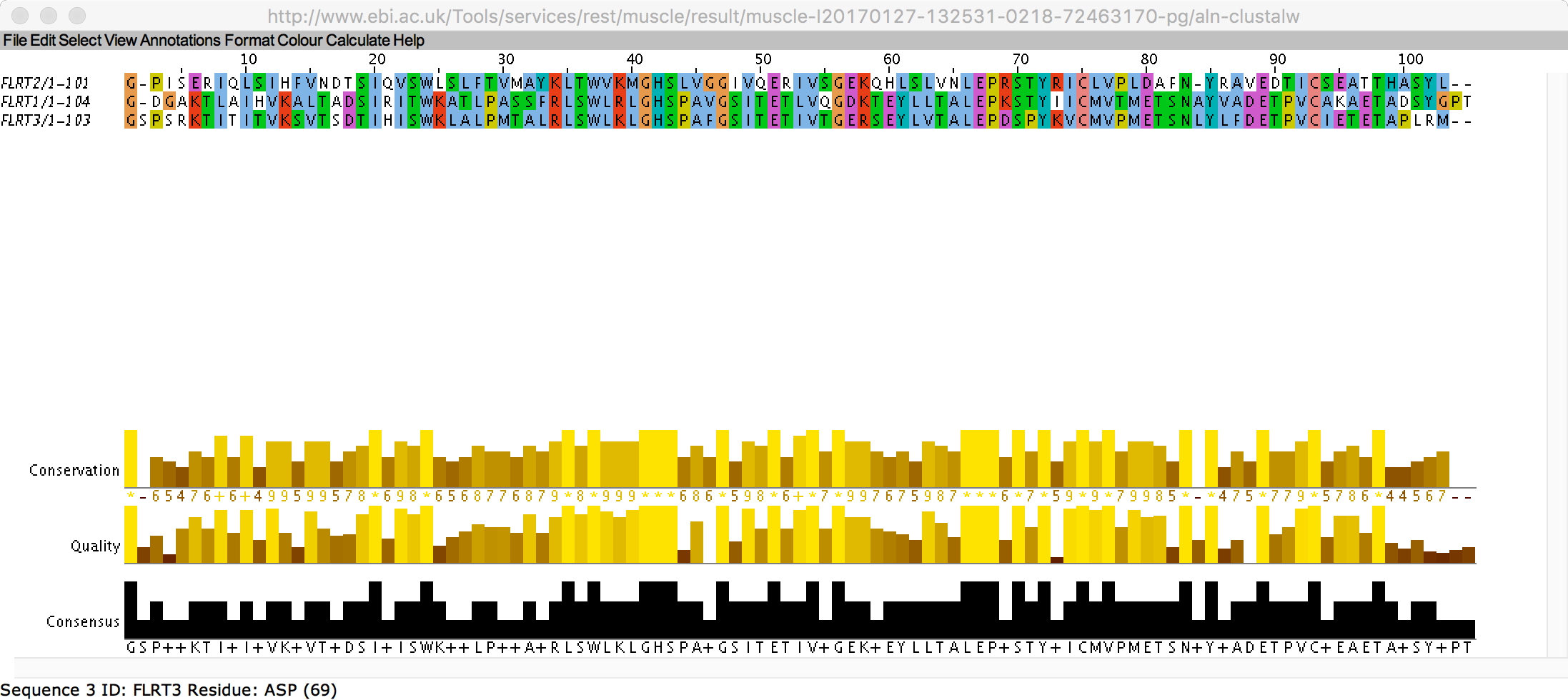


Amino acids sequences of FLRT-FnIII domains were aligned using MUSCLE (ebi.ac.uk). One notable difference is that the two cysteines (^75^Cys-^92^Cys) in the FLRT2-FnIII domain are spanned by a shorter sequence compared to FLRT1 and FLRT3, which have an extra amino acid between their ^75^Cys-^93^Cys.

Figure S3

FLRT1


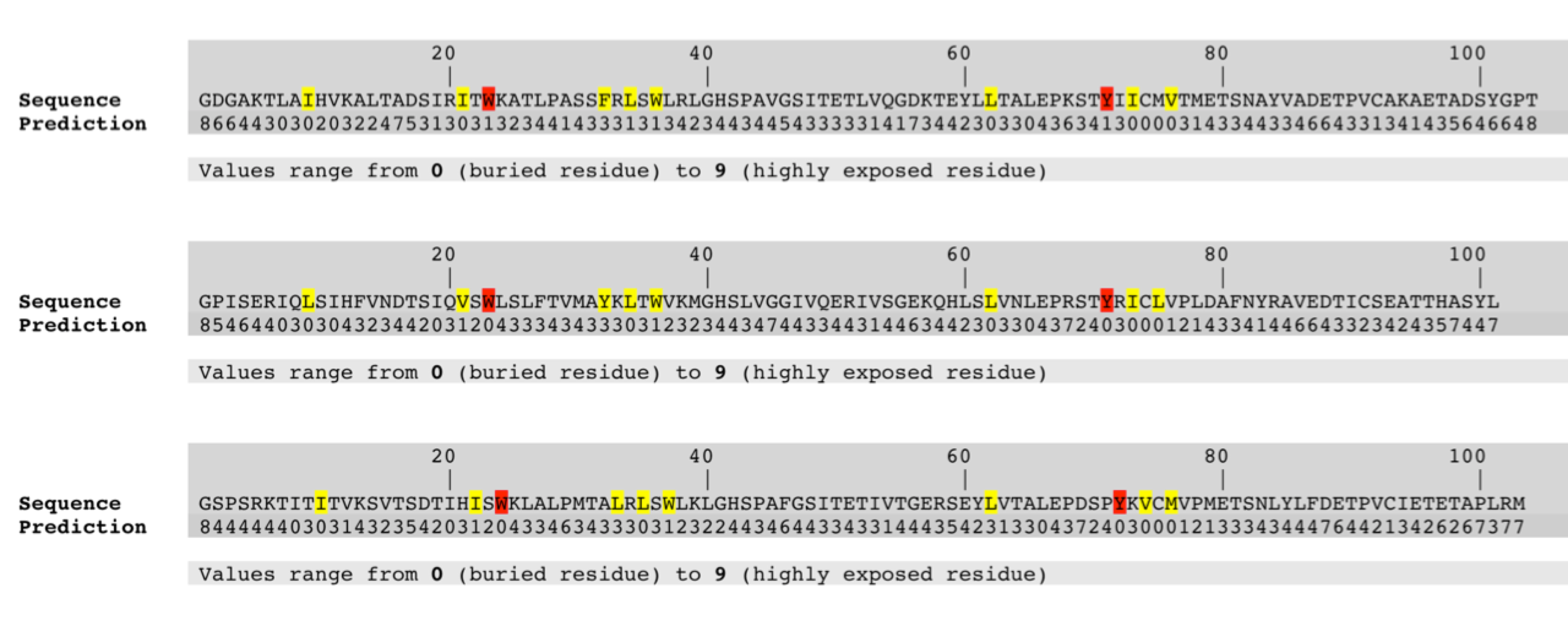


FLRT2

FLRT3

Sequences for FLRTs FnIII with I-TASSERs prediction of how buried each residue are. Values from ranging from 0-9 with 9 as highly solvent exposed. Characteristic hydrophobic patterns are observed for FnIII domains, and are colored in yellow and red. Residues highly conserved in FnIII domains are colored in red.

Figure S4

***
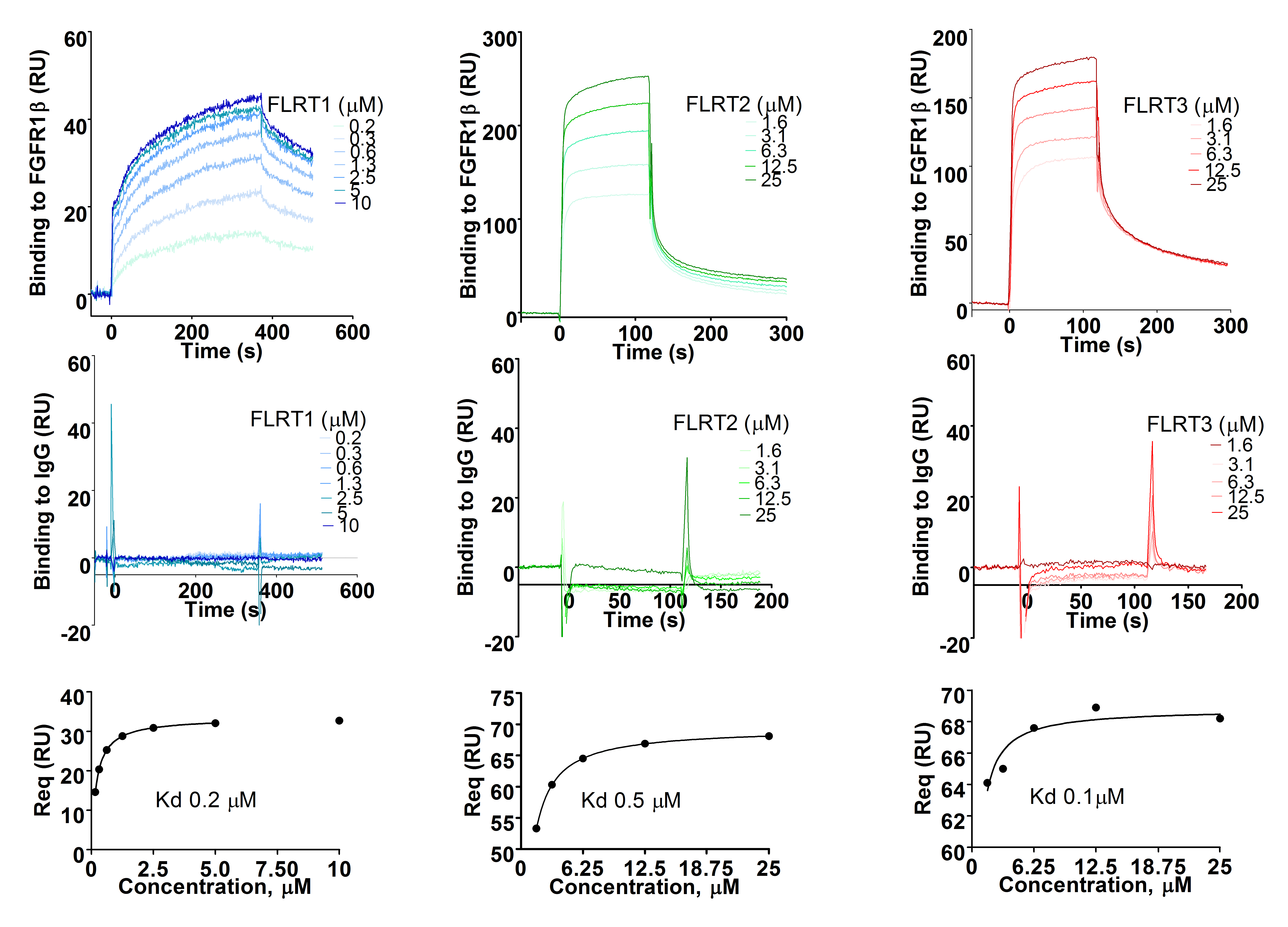
***

Surface Plasmon Resonance (SPR). SPR experiments verified *in vitro* binding between FGFR and FLRT-FnIIIs. A rough estimate of K_d_ for the interaction between FGFR1 and the different FLRT-FnIII domains were performed using a steady state model. It must be stressed that these K_d_s should be taken as preliminary, as the experiments were only performed twice. The three columns represents FLRT1- (first, blue), FLRT2- (second, green) and FLRT3- (third, red) FnIII domains flowed over immobilized FGFR1β (row 1), immobilized IgG as negative control (row 2) and roughly estimated K_d_s (row 3)
